# Workplace Violence Against Primary Care Clinicians: A Narrative Review

**DOI:** 10.1007/s11606-024-08850-3

**Published:** 2024-07-08

**Authors:** Nicholas D. Tyau, Kristin A. Swedish, Hector R. Perez

**Affiliations:** Department of General Internal Medicine, Montefiore Medical Center, Bronx, NY USA

**Keywords:** adult, physicians, general practice, primary health care, primary care, occupational hazard, violence, workplace violence/psychology, workplace violence/statistics, aggression, harassment, health care workers

## Abstract

Workplace violence (WPV) is a commonly reported occupational hazard in healthcare and its prevalence is increasing. WPV occurs in all types of practice settings, but little is known about WPV in primary care settings in the United States (US). Because primary care practice settings differ from the inpatient settings, further examination of WPV in primary care is warranted. Our objective was to summarize the available literature highlight important gaps. We conducted a search using Pubmed and OVID for US studies of WPV in US-based adult primary care practices. Studies including only pediatric populations were excluded. Due to the lack of available literature conducted in US primary care settings, we expanded our search to include international studies. We identified 70 studies of which 5 were US based. Due to the lack of significant numbers of US-based studies, we opted to conduct a narrative review of all available studies. The evidence shows that WPV is a common occurrence in primary care settings in many countries and that the majority of primary care clinicians have experienced at least some form of non-physical violence in their careers. Most of the studies conducted were cross-sectional in design and reported on both non-physical and physical forms of WPV. There was not a consistent trend between genders in experiencing the major forms of WPV, but women were consistently more likely to be subjected to sexual harassment. Potential root causes for WPV could generally be categorized as patient-level, clinician-level, clinical encounter specific, and operational root causes. While most WPV was found to be non-physical, it still had significant emotional and job-related impacts on clinicians. These troubling results highlight the need for further studies to be conducted in the US.

## INTRODUCTION

Workplace violence (WPV) is defined by the World Health Organization (WHO) as “incidents where staff is abused, threatened, or assaulted in circumstances related to their work, including commuting to and from work, involving an explicit or implicit challenge to their safety, well-being, or health”.^1^ In the United States, the healthcare industry experiences high rates of injury due to WPV. Persons working in the healthcare industry are five times more likely to be injured due to WPV than workers in other service industries such as finance, food and beverage, and retail.^2^ Moreover, WPV against healthcare workers is increasing, with incidence rates of non-fatal workplace violence almost doubling from 6.4 to 10.4 per 10,000 workers from 2011 to 2018.^3^

Despite these distressing numbers, little is known about WPV in primary care. Studying WPV in primary care is important because primary care is the foundation of the US health care system and is a platform for continuous, person-centered, relationship-based care that considers the needs and preferences of the patient and their community.^4^ Primary care visits are the most common form of medical care delivered in an office setting. In 2008, there were approximately 956 million office visits with 51% which were in primary care settings.^5^ Moreover, primary care visits are intimate encounters designed to create a safe space for clinicians and patients to establish a confidential line of trust and communication. Tragically, primary care remains underfunded and understaffed as a specialty, receiving only 5% of all health care spending dollars.^4^ These challenges may increase safety risks for primary care clinicians. Primary care clinicians serve as gatekeepers for specialty care and to other psychosocial services. Primary care clinicians see more patients and are often the managers of most of a patient’s medications.^6^ This role as a gatekeeper could place clinicians at an increased risk of conflict when expectations are not met regarding referral to services or prescribing medications.

To summarize the existing literature, we conducted a narrative review of WPV in primary care settings in the US. Specifically, we sought to explore the following regarding WPV: (1) prevalence, (2) types, (3) mechanisms, (4) causes, and (5) effects.

## METHODS

We conducted a narrative review of the literature related to workplace violence of primary care clinicians. For classifications of WPV in this review, we defined WPV as violence perpetrated by a customer or patient against an employee. We used the National Institute of Safety and Health definition of Type 2 WPV to develop our review criteria. Type 2 WPV is violence towards an employee by a client, family member, or visitor. Separately, we organized WPV into two categories: (1) physical, defined as pushing, spitting, property damage, punching, kicking, or sexual assault; and (2) non-physical defined as verbal insults, racist/bigoted comments, online threats/insults, verbal threats, bullying, sexual harassment, intimidation, and stalking.

To find articles, we conducted a search without any time parameters of PubMed and Ovid for articles on WPV against clinicians in adult primary care using the following MESH terms (“ambulatory care OR ambulatory care facilities” OR “outpatients” OR “family practice OR general practice OR primary care”) AND (“physician” OR “doctor” OR “general practitioner” OR “nurse practitioner” OR “physician assistant”) AND (“violence” OR “harassment, non-sexual, OR sexual harassment OR bullying OR “aggression” OR “crime” OR “crime victims” OR “abuse” OR “intimidation” OR “threat”) AND (“workplace violence” OR “occupational violence”). Additional studies were found through reference lists of included studies. Studies were selected for further analysis after reviewing the abstracts. Our inclusion criteria were (1) studies in English; (2) internal medicine, family medicine, or general practice clinicians (including physicians, nurse practitioners, and physician assistants) must be at least part of the study population; and (3) US-based setting. Our exclusion criteria were (1) exclusively inpatient settings, (2) exclusively overnight or settings where a clinician visits a patient’s home, and (3) exclusively pediatric populations in the practice. Our initial query yielded only one article about WPV in primary care settings in the US and five studies conducted in the US but not exclusively studying primary care clinicians. As such, we expanded our initial inclusion criteria to also include international studies of WPV.

To summarize article content, we used deductive thematic analysis to group articles into pre-defined areas of emphasis. Our pre-defined areas of emphasis included (1) prevalence of WPV, (2) types of WPV, (3) mechanisms of WPV, (4) causes for WPV, and (5) effects of WPV. One author (NT) reviewed all abstracts and articles and discussed findings with another author (HP), where consensus was reached on inclusion and exclusion of articles within themes. As study design and geographic area differed among included studies, no meta-analysis was conducted on findings, and instead data is presented narratively. In our analysis, we focused on findings that pertained to primary care clinicians (physicians, nurse practitioners, physician assistants) when possible; however, some studies did not conduct specific analyses on these populations.

## RESULTS

### Types of Surveys

After applying our expanded inclusion criteria, we selected 70 studies of which 60 were cross-sectional, two were repeated cross sectional, four were prospective studies, three were qualitative studies, and two were mixed methodology studies with both cross-sectional and qualitative components. Only five studies were conducted in the United States;^7–11^ two of which examined WPV among primary care clinicians. One of the studies involved only adult primary care clinicians.^7^ The other study Internal Medicine residents and assessed WPV in both the inpatient and outpatient settings.^11^ Two other US-based studies examined WPV clinicians in multiple specialties including physicians, nurses, and nurse’s assistants.^8^ One US-based study examined WPV experienced by women physicians in multiple specialties, but did not specify specific WPV incidence between specialties.^9^ The remainder, all international studies, were conducted in multiple countries including Barbados, Pakistan, Saudi Arabia, Japan, Turkey, Israel, Brazil, Belgium, Bulgaria, Lebanon, Portugal, South Africa, Thailand, Australia, Sweden, Serbia, New Zealand, China, Spain, Poland India, Italy, Canada, Congo, UK, Rwanda, Ireland, Croatia and Herzegovina, Germany, and Lithuania. Within studies, the sample sizes of clinicians studied ranged from 28 to 3514.^12,13^

### Types of Violence

Definitions of WPV varied among included studies, reflecting that terminology is often inconsistently applied to episodes of WPV. Most studies were in agreement with the definition of physical violence because it requires physical contact. On the other hand, definitions of non-physical violence were more variable. Ten studies labeled non-physical violence as simply verbal abuse.^12,14–22^ The remaining studies described a wide variety of behaviors in their definitions of non-physical violence, including verbal insults, racist/bigoted comments, online threats/insults, verbal threats, bullying, sexual harassment, intimidation, and stalking.

### Frequency of Violence

Thirty-three studies reported non-physical WPV to be more commonly experienced than physical WPV.^9,14,20,21,23–51^ The most common form of non-physical WPV reported was verbal abuse. The prevalence of verbal abuse within the prior 12 months ranged from 44 to 64% among outpatient clinicians.^25,34,36,37,39^ Some form of verbal abuse was noted in 60.7% of respondents in the one US-based primary care study.^7^ In contrast, the prevalence of physical violence within the prior 12 months in primary care settings was much less common, ranging from 0.5 to 15.9%.^25,29,32,38,39^

Since most of the studies were cross-sectional in design, there are limited data about trends in WPV in primary care settings. One prospective study and two repeated cross-sectional studies identified in the literature did not yield consistent results. A Spanish prospective study from 2010 to 2015 showed a statistically significant decrease in WPV from 20.2 to 15 per 10,000 clinicians over the 5-year period (*p* < 0.0001).^46^ One repeated cross-sectional study from Norway among randomly selected clinicians reported no significant difference in experiencing threats of violence from 1993 to 2014.^19^ Another repeated cross-sectional study from Italy that administered sequential surveys from 2005 to 2009 among outpatient healthcare staff found no change in physical violence and a decrease in non-physical violence over the 4-year time period.^40^ One US-based study noted an increase in incident rate from 4 to 6 per 10,000 worker-months between 2012 and 2014; however, this study did not specify trends in primary care settings.^8^

### Gender

There are inconsistent conclusions about differences in WPV between men and women noted in the literature. Twelve studies found no difference in WPV between women and men. ^19,22,24,25,28,29,32,34,36,42,46,47^ However, seven studies found that women experienced more non-physical violence,^13,18,35,39,43,48,49^ and two studies that found women experienced more physical violence than men.^17,39^ One type of WPV, sexual harassment, was shown in ten studies to consistently affect women more than men.^9,17,35–37,39,50–53^ In contrast, five studies noted that male clinicians were more likely to experience non-physical WPV ^12,14,32,51,54^ while 14 studies noted that men were more likely to experience physical violence more than women. ^12,31,33,35,36,44,48–51,54–57^

### Root Causes of WPV

When clinicians from international studies were asked what they thought contributed to incidents of WPV, there were some common themes that emerged as potential root causes. We categorize these into patient-level root causes, clinician-level root causes, clinical encounter specific root causes, and operational root causes (Table 1).

**Table 1:** Tyau WPV Review Table

Authors, year, countries, journal	Country	Study design	Participants	Results	Suspected root cause
Abed et al. 2016 Occ. Med	Barbados	CS	O, S *N* = 102 • 44 primary care clinicians	-79% male clinicians, 19% female clinicians experienced WPV -67% verbal abuse (women more than men—include #s if available), 3% physical abuse -Adjusted OR found women 9 times more likely to experience WPV	N/A
Adhikari et al. 2022 J Nepal Health Couns	Nepal	CS	O, I, S, Sub *N* = 302 • 100 primary care clinicians	- 62.9% of all surveyed exposed to WPV - Verbal abuse, threats, and intimidation were the most common forms of WPV	PL: Alcohol/drugs, distrust of diagnosis, worsening condition/death of patient CL: delay in treatment
Ahmed et al. 2018 Ann Med and Surg	Pakistan	CS	O, I *N* = 524 clinicians • Though no specification on specialty, Discussion section mentions primary care clinicians in study group	- Women felt less safe than men (statistically significant difference) - Verbal abuse most common - Harassment also common	N/A
Al-Turki et al. 2016 J of Multi Disc Health	Saudi Arabia	CS	O, S *N* = 133 primary care clinicians	- 45.6% of those surveyed experienced WPV within 12 months - 99.2% of WPV was non-physical - Almost 1/3 of those felt WPV reduced their work performance—majority of HCW were either unsatisfied for very unsatisfied with the consequences of reporting - Clinicians were the third highest group affected	OL: lack of penalty for offender, long wait times CL: misunderstanding, unmet service demanding VL: overcrowding PL: reaction to injury
Alsaleem et al. 2018 J of Fam and Comm Med	Saudi Arabia	CS	O, I *N* = 321 clinicians • 154 primary care clinicians	- 78 (24.3%) primary care clinicians exposed to WPV - Nurses more likely to experience WPV than clinicians - 32.9% verbal, 11.1% physical, 55.9% both verbal and physical - 58% of those exposed said it affected job performance - No statistically significant difference between violence against men vs women - Primary care clinicians experienced more violence than hospital clinicians	PL: lack of pt education, culture/personality OL: long wait time, staff shortages, lack of security VL: overcrowding
Alsmael et al. 2020 Int J of Gen Med	Saudi Arabia	CS	O, S *N* = 360 healthcare workers • 84 primary care clinicians	- 47% experienced WPV in 12 months - 90% verbal, 34% intimidation - No significant difference between genders	OL: lack of penalty for offender, long wait times VL: overcrowding CL: inadequate training, unmet service demand, miscommunication
Arimatsu et al. 2008 J of Occ Health	Japan	CS	O, I, Sub *N* = 698 • 167 internal medicine clinicians • 45 respondents worked in a clinic	- 32% of clinicians experienced at least one episode of verbal violence - 11 clinic-based internal medicine clinicians experienced verbal violence in last 6 months	N/A
Aydin et al. 2009 J of Int Viol	Turkey	CS	O, I *N* = 522 clinicians • 256 primary care clinicians	- 81% of clinic-based clinicians experienced WPV - 89.3% of WPV was verbal - Verbal and sexual violence more common in women - 15% of respondents did nothing to defend themselves because they ignored it or were afraid of consequences	CL: unmet prescription demand, unmet work letter demand, dissatisfaction with treatment OL: long wait times, medical expenses PL: alcohol/drug misuse, work stress
Baykan et al. 2015 Int J of Occ Safety and Erg	Turkey	CS	O, I, Sub *N* = 597 • 124 primary care clinicians	- 86.4% of surveyed clinicians experienced at least one type of violence in their careers - 96.8% experienced verbal violence - 26.8% experienced physical violence - 59.5% witnessed physical violence, 86.6% witnessed verbal violence against other healthcare workers - No significant difference between genders for any type of violence; however, men more likely exposed to physical violence and women more likely to be exposed to verbal	PL: excessive demands, blaming physicians for problems CL: miscommunication OL: workload, insufficient resources, insufficient personnel
Carmi-Iluz et al. 2005 BMC Health Serv Res	Israel	CS	O, I, Sub *N* = 177 • 85 primary care clinicians	- 48 (58.5%) of community clinicians experienced verbal abuse - 28 (25.5%) stated that violence had a detrimental effect on their lives • Seventeen of those who said that violence had a detrimental effect on their lives (60.7%) were women (*p* < 0.05) • 17 community-based clinicians (36.2%) said that violence had a detrimental effect on their life	OL: long wait times PL: disagreement with physician, dissatisfaction with treatment CL: declines to give work letter
Çevik et al. 2020 Int J of Health Plan and Man	Turkey	CS	O, I, Sub *N* = 948 • 367 primary care clinicians	- 83.3% of clinicians experienced violence, verbal most common - 33% occurred at primary care office	OL: long wait times CL: declines to prescribe medication
da Silva et al. 2015 Soc Psych and Psych Epi	Brazil	CS	O, S *N* = 2940 • 217 primary care clinicians	- Examined depressive symptoms amongst primary care clinicians who witnessed WPV - Clinicians were only 7.4% of those surveyed - Most participants were women - Insults were most common form of violence, followed by witnessing violence	N/A
De Jager et al. 2019 BMJ Open	Belgium	CS	O, I *N* = 3726 • 1,490 primary care clinicians	- 84% had experienced aggression within their career • 37% within 12 months of the survey - 77% verbal abuse - 24% physical abuse - More women than men experienced aggression - Women experienced non-verbal violence more than men - Men experienced more physical violence	N/A
Demeur et al. 2018 BJGP Open	Belgium	CS	O *N* = 247 primary care clinicians	- 79.8% experienced aggression in the last year - Verbal aggression 89.8% - Psychological aggression 21.1% - Physical aggression 20.6% - Men felt safer than women	OL: long wait times CL: unmet patient demands PL: disagreement with clinician’s care plan
Di Martino et al. 2002 Synthesis Report	Multi-national	CS	O, I, Sub, S *N* = 400–1100 (study populations from several countries) • 40–140 clinicians	- Physical violence 3–17% - Psychological (non-physical) 27.4–67% - Bullying 10.7–30.9% - Racial/sexual harassment 0.8–8%	OL: lack of security, inadequate policies, lack of resources PL: personality traits of patients, substance use VL: management style Other: social/economic situation of country, health care reform, work organization, lack of procedures, crime, interpersonal organization issues, poor policing
Duan et al. 2019 Heal and Qual of Life Out	China	CS	I *N* = 444 internal med clinicians	- 66.19% experienced WPV - 65.3% verbal violence - No difference between genders - WPV associated with increased burnout turnover intention - Less job satisfaction	N/A
Dyrbye et al. 2022 JAMA Open Net	United States	CS	O, I, Sub *N* = 6512 • 435 primary care clinicians	- Women clinicians significantly more likely to receive racially offensive, discriminatory comments (verbal violence), unwanted sexual remarks or advances - 29.4% racial verbal abuse - Ethnic minority clinicians had higher racial discrimination score	N/A
El-Gilany et al. 2010 J of Intpers Viol	Saudi Arabia	CS	O, S *N* = 1091 • 211 primary care clinicians	- 27.7% of workers exposed to any type of violence - 92.1% exposed to emotional violence - 52.4% exposed to verbal abuse	VL: overcrowding, language barrier CL: unmet service demand, miscommunication OL: lack of penalty for the offender, lack of security, hot environment, understaffing PL: illiteracy, mental illness
Elston et al. 2002 Soc of Health and Ill	UK	CS	O *N* = 697 primary care clinicians	- 75% experienced verbal abuse - 10% experienced physical violence	PL: mental illness, substance use
Eneroth et al. 2017 Scan J of Prim Care	Sweden	CS	O *N* = 283 primary care clinicians	- Foreign-born clinicians statistically significantly more likely to experience threats than native-born - Foreign-born clinicians were more likely to report intention to leave clinical practice (statistically significant)	N/A
Feng et al. 2022 Hum Res for Health	China	CS	O *N* = 4376 primary care clinicians	- Verbal abuse the most common - Women in rural areas less likely to experience any type of violence - Women with high patient loads were more likely to experience WPV	OL: long wait times PL: substance use, mental illness, dissatisfied with service, medical costs CL: unmet service need
Fisekovic et al. 2015 Euro J of Pub Health	Serbia	CS	O, S *N* = 1526 • 438 primary care clinicians	- 52.6% experienced WPV, 18.3% physical WPV - No difference between genders - Nurses were attacked more than clinicians	N/A
Forrest et al. 2011 Med J Aus	Australia	CS	O *N* = 804 primary care clinicians	- 57.5% received verbal abuse - Male clinicians most likely to experience property damage - Female clinicians most likely to be sexually harassed - WPV affected emotional well-being	N/A
Frank et al. 1998 Arch Int Med	United States	CS	O, I, Sub *N* = 4357 • 936 primary care clinicians (women only)	- Gender-based harassment 13–33% amidst primary care clinicians - Sexual harassment 8–20% amidst primary care clinicians	N/A
Frieden et al. 2015 MMWR	United States	CS	I *N* = 2034 WPV injuries reported	- Trends WPV in healthcare in US increasing over 2 years - 99% of WPV injuries reported were physical WPV - Rates highest for nurses	N/A
Gale et al. 2006 New Zeal Med J	New Zealand	CS	O *N* = 1205 primary care clinicians	- Prevalence of all non-physical forms of abuse (verbal, threat, intimidation, sexual harassment): 41% - 15.4 per 100 for verbal threat 0.76 per 100 for assault requiring medical attention - Male clinicians more likely to be verbally threatened and assaulted - Female clinicians more likely to be sexually harassed	PL: mental illness, sexually inappropriate comments CL: declines sick letter/benefits letter, declines medication request
Gan et al. 2018 Am J of Pub H	China	CS	O *N* = 1015 primary care clinicians	- 62.2% experienced WPV within last year: 18.9% physical, 61.4% non-physical - Men with higher professional level and lower income more likely to experience physical WPV	N/A
Gascon et al. 2009 Int J of Env Health	Spain	CS	O, I, S, Sub *N* = 1826 • 206 primary care clinicians	- 11% victims of assault, 5% on more than one occasion - 64% victim of threats, intimidation, insults - Statistically significant differences were found between small hospitals/rural primary care facilities (9.5% and 11.3%) and large hospitals/urban primary care facilities (21.9% and 17.4%) • Physical violence seems to be associated with the size and complexity of the facility and its location - No significant difference found between genders - Only 3.7% of incidents were reported. These were all physical assault. None of the threats/insults were reported	OL: long wait times CL: declines to give letter for work, declines to prescribe a specific medicine
Hacer, Ali 2020. J of For and Legal Med	Turkey	CS	O *N* = 310 primary care clinicians	- 93.2% exposed to verbal abuse - 86.1% exposed to psychological violence - 22.6% exposed to physical violence - No significant difference in WPV found between genders	N/A
Heponiemi et al. 2014 BMC Health Serv Res	Finland	Pro	O, I, Sub Phase 1 *N* = 2841 Phase 2 *N* = 1515 • 328 primary care clinicians	- Bullying and job control were significant for intention to quit job - Physical violence and bullying were associated with lower levels of job satisfaction	N/A
Hills et al. 2012 Med J of Aus	Australia	CS Part of a large national study called Medicine in Australia: Balancing Employment and Life (MABEL)	O, I, Sub *N* = 9449 • 3514 primary care clinicians	- This study, based on MABEL population, examined reports of verbal/written or physical aggression - 70.6% of clinicians experience verbal or written aggression - 32% of clinicians experienced physical aggression within 12 months - Overall verbal/written aggression: men 69.1% vs women 72.6% - Physical aggression: 31.2% men vs 33.8% women - Primary care men experienced more verbal and physical aggression - International medical graduates more likely to experience WPV	N/A
Hills and Joyce. 2014 Med J of Aus	Australia	CS	O, I, Sub *N* = 9449 • 3514 primary care clinicians (same population as Hills 2012 study)	- This study, based on MABEL population, examined internal (within the work environment) aggression or external aggression - 27.2% experienced internal aggression - 67.6% experienced external aggression - Exposure to internal or external aggression negatively associated with job satisfaction, life satisfaction	N/A
Hills and Joyce. 2016 Ann of Occ Hyg	Australia	CS	O, I, Sub *N* = 9449 • 3514 primary care clinicians (same population as Hills 2012 study)	- This study, based on MABEL population, examined association between exposure to WPV and intention to leave patient care - Exposure to EPV was associated greater likelihood to reduce clinical workload or leave patient care - After adjusting for job satisfaction, life satisfaction, exposure to aggression was associated with being more likely to leave pt care within 5 years	VL: noisy, uncomfortable environment OL: long wait times
Hobbs 1991 BMJ	UK	CS	O *N* = 1093 primary care clinicians (82% male)	- 62.9% experienced some form of aggression, 14.6% said it was increasing - 91.3% verbal abuse exclusively	PL: substance use, mental illness OL: long wait times
Huang et al. 2020 BMJ Open	China	CS	O, I, S, Sub *N* = 87,988 healthcare workers (no description on characteristics or number of clinicians)	- Outcomes tracked as patient/visitor violence (PVV) - Increasing outpatient workloads increased risk of physical and non-physical PVV - Multiple linear regression analysis showed positive relationship between non-physical WPV and outpatient workload	N/A
Jankowiak et al. 2007 Adv Med Sci	Poland	CS	O, I, Sub *N* = 501 clinicians (no clear distinction which ones were primary care)	- 91% of outpatient affected by verbal aggression - 62% outpatient threatened	OL: staff shortages PL: stress
Johansen et al. 2017 BMJ Open	Norway	Repeated CS	1993 population: *N* = 2628 • 1271 general practice and internal medicine 2014 population: *N* = 1158 • 593 general practice and internal medicine	1993 vs 2014 - 52.6% vs 50.6% experienced threats - 25.3% vs 23.9% experienced physical violence - Statistically significant less WPV in 2014 compared to 1993 - No difference between genders at either time	N/A
Kader et al. 2021 Front in Psych	Bangladesh	Qual	O, I, Sub *N* = 165 (population determined by posts on a social media platform)	Themes noted, outlined in next column	CL: delayed treatment PL: “power practice” (intimidation of clinician), death of patient
Khan et al. 2021 BMC Health	Pakistan	CS	O, I, S *N* = 842 • 172 clinicians (though not defined which were primary care)	- 51% were exposed to violence in past year - 26% witnessed violence - Verbal abuse was the most common form (51%) - 23% experienced a combination of physical and verbal abuse - Effects of violence: 45% distressed, 5% severely distressed	CL: communication error, unmet expectations, human error PL: perceived substandard care OL: management failure
Koritsas et al. 2007 Brit J of Gen Prac	Australia	CS	O *N* = 211 primary care clinicians	- 57% of clinicians had experienced at least one episode of violence in last 12 months, verbal abuse most common - Women more likely to experience sexual harassment	N/A
Kunnath et al. 2023 Cureus	India	CS	O, I, Sub *N* = 1948 (Not defined how many worked in primary care settings)	- 65.6% faced some form of violence - 89.9% verbal abuse - 32.7% intimidation via gestures - 23.8% threats - 33.6% occurred in outpatient settings	N/A
Lindquist et al. 2020 Cureus	Myanmar	CS	O, S *N* = 63 • 25 primary care clinicians	- 47.6% experienced at least verbal or physical violence - 47.6% verbal abuse, 4.8% physical abuse - 56% were worried about WPV - 96.7% of those that experienced WPV were negatively impacted psychologically	N/A
Lowry et al. 2019 Southern Med J	United States	CS	O, I *N* = 88 internal medicine trainees	- Across both inpatient and outpatient settings - 41 (47%) of trainees experienced WPV - Type of threats/assaults: 78 events reported by 42 trainees: • 53% verbal • 23% emotional • 12% physical • 3% sexual	N/A
Magin et al. 2005 Med J of Aus	Australia	CS	O *N* = 528 primary care clinicians	- 63.7% experienced some form of violence - Most common: verbal (42%), property damage (28%) - 49% experienced low-level violence, 12.9% high level violence - Women were more likely to experience physical abuse, sexual abuse, stalking, harassment, verbal abuse, slander, and property damage	PL: substance use, mental illness
Magin et al. 2006 Fam Prac	Australia	Qual	O *N* = 18 primary care clinicians	Clinicians explored themes on how to deal with violence in the workplace: - Primary strategies: prevent aggression from occurring. Achieved via restricting where they practiced (avoid home visits or certain neighborhoods), reducing after hours work, discouraging certain categories of pt (opiate seeking) through disincentives, individual level strategies like personally vetting pts, discouraging violence by reducing solo physician scenarios - Secondary strategies: preventing aggression from escalating to more serious forms, interpersonal skills, physical retreat, acquiesce to demands, physical boundaries like counters - Tertiary: dealing with established violence. Self-defense (though participants largely had no plan)	N/A
Magin et al. 2008 Can Fam Phys	Australia	Qual	O *N* = 172 primary care clinicians (18 focus group members, 154 participated via written responses)	Themes listed in next column	PL: mental illness, substance use, dementia/delirium OL: long wait times CL: denial of care, improper training in de-escalation, lack of interpersonal skills, declining to write letter PL: poverty, unemployment Societal causes: loss of respect of authority, entitlement, degradation of mores and evolving culture of fear
Magin et al. 2011 Aust Health Rev	Australia	CS	O, S *N* = 125 • 46 primary care clinicians	- Study comparing WPV against primary care clinicians vs staff - 12-month prevalence of violence 59.3% for clinician, 74.6% for non-clinicians	N/A
Magnavita and Hepoinemi 2012 BMC Health Serv Res	Italy	Repeated CS	O, I, S, Sub *N* = 1166	- Three cross-sectional studies with 2-year intervals - 9.2% experienced physical aggression - 19.6% experienced verbal aggression - Men experienced verbal threats more than women - Clinicians and nurses most exposed to physical aggression and threats but it was not statistically significant	N/A
Malinauskiene and Einarsen 2014 Int J of Occ Med and Env Heal	Lithuania	CS	O *N* = 323 primary care clinicians	- Bullying: systematic and long-term exposure to aggression and social exclusion - Study also examined PTSD symptoms - Did not find differences in gender with regard to bullying or PTSD symptoms - 17.3% occasional bullying - 13% severe bullying - 15.8% PTSD symptoms - 11.8% bullying from pts - 26.6% bullying from superiors	N/A
Miedma et al. 2009 Can Fam Phys	Canada	Qual	O *N* = 48 primary care clinicians	- Groups were created based on selected demographic characteristics, these groups were meant to allow for more diversity of experience such as young/rural/female/French Themes - lack of respect: from patients and male colleagues, reported more amongst women - demanding pts: threatening clinicians due to not filling forms or scripts - severe abuse encounters: not common but when it happens, is frightening. reports of abuse from male colleague, stalking	CL: declining to fill form or script PL: substance use
Miedema et al. 2010 Int J of Fam Med	Canada	CS	O *N* = 774 primary care clinicians	- Tracking monthly incidence of WPV - 29% experienced WPV within last month - 59% of clinicians working in ED reported at least one incident within 1 month - 8% reported severe abuse consisting of stalking, assault, sexual assault, assault resulting in an injury - Most incidents were classified as “minor” (non-physical) - White clinicians statistically significantly more exposed to WPV than non-White clinicians	PL: mental illness
Miedema et al. 2010 Can Fam Phys	Canada	CS	O *N* = 774 primary care clinicians	- 98% reported at least one WPV encounter in career: 97% minor, 75% major, 39% severe - Most commonly disrespectful behavior - Women significantly more frequently bullied and sexually harassed - Men significantly more likely to be threatened, assaulted, property damaged	N/A
Muzembo et al. 2015 J of Occ Health	Democratic Republic of Congo	CS	O, I, S, Sub *N* = 271 clinicians (though not specified how many primary care)	- 80.1% of all clinicians experienced at least one instance of WPV in last year - 69.1% verbal aggression, 12.1% harassment, 6.9% physical violence - Of the clinicians reporting harassment 63.6% were women reporting sexual harassment - Men more likely victims of physical violence, women more likely to be harassed	PL: disrespect of medical staff
Ness et al. 2000 BMJ	UK	CS	O *N* = 380 primary care clinicians	- Women experienced more verbal abuse - Men experienced more physical abuse	PL: mental illness, substance use
Nevo et al. 2019 BMC Health Serv Res	Israel	CS	O, I *N* = 145 • 82 primary care clinicians	- 49% of primary care clinicians experienced verbal abuse within last year	OL: long wait time CL: declined to prescribe medication PL: dissatisfied with treatment
Newman et al. 2011 Hum Res for Health	Rwanda	CS and Qual	O, I, S, Sub *N* = 12 clinicians (though not clear which were primary care)	- 39% of clinicians reported experiencing at least one form of workplace violence in the twelve months prior to the study: • 27% of respondents had experienced verbal abuse • 16% were bullied • 7% encountered sexual harassment • 4% were physically assaulted - Women sexually harassed more than men	N/A
Nøland et al. 2021 JAMA Open Network	Norway	Pro	O, I, Sub *N* = 893 medical students	- Two cohorts followed for 15 then 20 years - 21–30% of the respondents were clinicians - No difference between genders in regard to threats of violence - During the first two follow-ups, men were statistically significantly more likely to experience acts of violence, but over time this decreased significantly - Young men were at highest risk—Psychiatry highest risk specialty	N/A
O’Connell and Bury. 1997 Fam Med	Ireland	Pro	O *N* = 354 primary care clinicians	- Overall incidence 21% - Those with more patients and less staff more likely to experience WPV - No difference between genders - Non-physical violence most common (verbal + threats)	N/A
ONAM 2018 BMC Res Notes	Spain	Pro	O, I * N* = 2401 • 1267 primary care clinicians	- Data collected over 6 years - Cumulative incidence showed a decrease of WPV over the study period - No gender difference for physical or psychological injury - More aggressions in primary care 54%	PL: mental illness, substance use, disagreement with treatment plan CL: declines to prescribe medication, declines to write letter or sick leave form OL: long wait times
Ozdamar et al. 2022 Fam Prac	Turkey	CS	O, I, S, Sub *N* = 701 (460 clinicians but does not specify which are primary care)	- Pre- and post-COVID study though cross-sectional study in design - 54.1% had at least one episode of violence - Verbal abuse was most common - Clinicians working in family health centers and internal medicine more likely to be exposed to violence relative to other workers - Exposure to violence more common among clinicians than staff, more common among women compared to men - Those exposed to violence had increased burnout, this got worse during pandemic period - Exposure to violence decreased during the pandemic	N/A
Parker et al. 2010 National Report	Australia	Stage: 1 Literature review Stage 2: Stakeholder consultation Stage 3: Qualitative focus groups with clinicians Stage 4: Online/paper based surveys	O Qualitative *N* = 78 staff *N* = 20 primary care clinicians Online survey *N* = 178 primary care clinicians Paper survey *N* = 781 primary care clinicians	Qualitative findings - Designed to explore staff experiences with WPV - Stakeholders felt their organizations were not proactive in advocating for harm minimization or post incident support; however, some groups felt that at a work environment level, measures were starting to be implemented - Barriers preventing implementing measures included cost of renovation or cost of alarms, some practices ignoring the problem Online survey results - 95% experienced some form of verbal aggression, 70% within last year - Male clinicians experienced more verbal aggression, stalking - Female clinicians more likely to experience sexual harassment - 56% experienced physical aggression as property damage - 26% ever experienced sexual harassment - Aggression affected emotional well-being Paper survey results - 88% experienced some form of verbal aggression, 58% in last year - Male clinicians experienced more verbal aggression, property damage than female - Female clinicians were experienced stalking - Female clinicians more likely to experience sexual harassment - Aggression negatively affected emotional well-being	OL: long wait times, inability to see specific doctor, lack of security CL: declining a certain medication
Pendergrast et al. 2021 JAMA	United States	CS	O, I, Sub *N* = 464 clinicians (though not defined by specialty)	- Assessed social media harassment - No gender difference between personal attacks - Women were statistically significantly more likely to be sexually harassed online than men	N/A
Phillips and Schneider 1993 NEJM	Canada	CS	O, I, Sub *N* = 417 primary care clinicians	- 77% of respondents were sexually harassed during their career - Majority of cases, perpetrator was male - Responses to harassment: fear, anger, amusement - Effects of mobbing: lack of motivation, loss of confidence, depressive symptoms, sleeplessness, anxiety	N/A
Rincon et al. 2016 Rev Esp Salud Publica	Spain	CS	O, I, S, Sub *N* = 1157 • 561 family physicians	- 48.4% family physicians assaulted - 75.2% of assaults were verbal insults - Women assaulted more than men	PL: dissatisfied with care plan OL: long wait times CL: declined to prescribe medication
Saeki et al. 2011 J of Occ Health	Japan	CS	*N* = 588 long questionnaire *N* = 170 short questionnaire includes internal medicine	- Data by specialty is reported as number incidents rather than specific number of physicians - 15.7% annual incidence - Verbal abuse and threats most commonly reported - 53.9% experienced WPV at least once in their careers - Women more at risk than men	
Sansone et al. 2017 Prim Care Comp J of Clin Psych	United States	CS	O *N* = 61 primary care clinicians	- 60.7% were verbally bullied to write a prescription - 45% were verbally bullied to fill out a form - 85% resulted in cursing out staff	CL: declined to fill out form, declined to fill out medication, declined to make a referral OL: lack of security
Sari et al. 2023 PLoS ONE	Turkey	Repeated CS	O,I, S *N* = 135 • 84 clinicians (though not clear which ones were primary care)	- 71.9% verbal WPV - 28.1% physical WPV - No significant difference between genders - Clinicians accounted for 62.3% of reports - Most cases were reported in outpatient clinics	PL: non adherence with procedures CL: miscommunication OL: long wait times
Tuncel et al. 2009 J of Intpers Viol	Turkey	CS	O, S *N* = 506 • 123 primary care clinicians	- 47.2% reported any type of violence within last year. Verbal abuse most common - All types of violence except physical were more common in females - Younger clinicians experienced statistically significantly more violence than older clinicians - Less experienced clinicians experienced statistically significantly more violence than older clinicians	N/A
Vorderwülbecke et al. 2015 Deus Arz Int	Germany	CS	O *N* = 831 primary care clinicians	- Majority felt safe or very safe - 91% experienced some form of aggression at some point in their career. 73% within 12 months - Mildly/slightly aggressive incidents were the most common 79% in last 12 months - 81% moderate aggression - 23% severe aggression - Verbal insults most common - Sexual harassment more common in female clinicians	N/A
Yang et al. 2019 BMJ Open	China	CS	O, I, S *N* = 4862 • 1954 responses from clinicians • 1480 responses were from primary care providers	- Yi Nao: organized form of WPV that are planned against a facility or workers - 40.4% experience or witnessed Yi Nao - 4.6% experienced physical WPV - 17.4% experienced threats - Victims experienced flashbacks, hypervigilance, less trust in patients - Male workers more likely to experience Yi Nao, physical violence, threats - Hospital workers more likely to experience violence compared to primary care setting	N/A

*O* outpatient clinicians; *I* inpatient clinicians; *S* other clinical staff including nurses, receptionists, administrators; *Sub* other specialist clinician (non internal medicine, non family medicine); *CS* cross-sectional; *Qual* qualitative; *Pro* prospective; *PL* patient level root causes; *CL* clinician level root causes; *VL* clinical visit root causes; *OL* operational level root causes

Patient-level root causes can potentially escalate tensions and could precipitate a violent incident. Ten studies noted that patients’ substance dependence and/or psychiatric conditions led to violent incidents.^7,14,24–26,33,46,51,58,59^ Some of these conditions included anxiety and psychosis, but clinicians also noted that some patients had high levels of stress, had previous violent episodes, or had personality traits such that made them quicker to become angry.^16,26^ Two studies noted that violent incidents were results of reactions patients had when they received bad news such as a serious illness or death of a family member.^25,58^ Two studies mentioned that patients’ “lack of education” or “low educational level” contributed to episodes of WPV. ^26,28^ Separately, lower health literacy was mentioned as a potential root cause.^25^ While any of these are potential contributors, a systematic categorization of patient-level root causes has not yet been developed.

Clinician-level root causes also contributes to tensions that can lead to WPV. Two studies noted that clinicians felt that stress due to concerns about job security, poor working conditions, a lack of resources, lack of training, clinician error, and understaffing contribute to WPV.^16,26^ Other causes, such as clinician personality traits or communication style, could contribute to tensions as well.^27,57,59^

While individual patient or individual clinician root causes can contribute to WPV, clinical encounter specific factors can also lead to several unique root causes. Seven studies cited unmet expectations for care as the root cause of an incident.^22,25,44,46,51,54,60^ Six studies noted clinicians not meeting patient expectations, ^27,35,42,46,51,54^ including a physician’s refusal to complete a work letter or refusal to prescribe a particular medication. Seven studies noted a disagreement in treatment plan,^15,27,35,42,44,45,61^ and three studies noted an error in communication as a clinical encounter specific factor.^22,25,26^

Clinicians also reported that operational root causes can contribute to WPV. Ineffective workflows within an institution could lead to long workdays and understaffing, which contributes to increases in wait times.^26,28^ Additionally, staff shortages can lead to long waiting times, which has been listed by thirteen studies.^15,18,25,27,28,32,35,42,46,54,58,59,61^ Other operational problems include underdeveloped or inadequate safety policies or procedures for WPV. Clinicians noted in four studies that inadequate policies were a contributor to incidents of WPV. ^22,25,26,59^ Additionally, two studies also stated that clinicians felt that a lack of consequence for aggressors encouraged further WPV.^25,26^

### Effects of Violence on Clinicians

Even though the vast majority of WPV is nonphysical, it has a significant effect on its victims. The effects can influence people psychologically and studies to date have found experiences of WPV are associated with symptoms of anxiety, depression, post-traumatic stress disorder, hyper vigilance, suspicion, burnout, distress, poor well-being, and work dissatisfaction in clinicians.^22,36,54,62–64^ Additionally, three studies have shown WPV leads to a reduction in work performance.^25,26,64^ Clinicians from two studies have also noted negative effects on their quality of life outside of work.^15,22^ Ultimately, these effects have been associated with increased absenteeism and increased turnover.^41,65^

Lack of institutional support exacerbates effects of WPV on primary care clinicians. Two studies note that that a lack of institutional support in response to WPV can leave clinicians feeling vulnerable and unsafe at work.^30,47^ Clinicians may also feel unsupported if they do not have the proper training to deal with WPV. They may feel frustrated because of a lack of de-escalation training, and because they may feel they cannot discharge violent patients from their practice.^25,66^ If there is no confidence in their employer for support, this may lead to decreased motivation to report concerns or incidents, exacerbating symptoms of burnout and work dissatisfaction.^6,18,62^. Several studies demonstrate that many clinicians do not report episodes of violence even when there are operational causes due to perceived indifference by their employer.^29,54^ Other researchers have concluded that empowering primary care clinicians to report may increase confidence and comfort, and may actually lead to lower incidence of WPV.^23^ Even if reporting does not result in lower incidence of WPV, more comprehensive data can spur employers to prioritize the problem.^46^

## Conclusion

We found that data for WPV in primary care setting in the US was sparse, though the one study that we did find was consistent with the prevalence of WPV reported in international primary care studies, ranging from 10 to 75%.^67^ Non-physical violence, especially verbal abuse, was by far the most common type of violence experienced by primary care clinicians. While there were inconsistent findings with respect to how WPV may differ by gender, women were significantly more likely to be targets of sexual harassment. Primary care clinicians named numerous possible root causes for WPV, which could be categorized into patient level, clinician level, clinical encounter specific, and operational root causes. Even though most WPV is non-physical, there can be profound negative psychological and emotional effects on clinicians that can directly affect work performance and may lead to increased turnover.

This review highlights the need for increased investigation into WPV in the US. We found that the majority of international studies are small and cross-sectional, and depend on adequate reporting. Studies using longitudinal designs are scarce. Two existing longitudinal studies from Israel and Norway thus far have not shown increases in violence over time.^15,19^ Additional longitudinal studies from other countries including the United States are needed to better understand how WPV in primary care evolves over time. One significant challenge in data collection is the cultural belief within healthcare occupations that being verbally accosted, threatened, or even physically assaulted is “part of the job.” As a result of this attitude and because of a lack of confidence in safety policies or post incident support, the true extent of WPV remains under reported and therefore poorly understood.^18,29,40^ Allowing this problem to continue will only perpetuate the negative effects of WPV on clinicians. Allowing even verbal abuse to continue not only has profound psychological and emotional impact, but can have harsher downstream consequences since verbal assault is a risk factor for physical assault.^68^

More primary care–specific WPV data will allow for stronger national interventions to support clinicians and other clinical staff at any stage surrounding a violent incident. There are initiatives in other medical specialties, such as emergency medicine, to develop protective policies. In the hospital, advocacy from clinicians has led to new Joint Commission standards as of January 2022, which included guidance for employers aimed at reducing WPV in the hospital setting. These standards recommend processes for monitoring workplace conditions, educating clinicians, and creating a culture of safety. It has also led to proposed legislation such as the “Safety from Violence for Healthcare Employees (SAVE) Act,” which establishes federal penalties for violence against hospital-based healthcare workers. While some of these protections benefit clinicians from every specialty, no specific initiatives have been developed for outpatient settings. Additionally, more primary care–specific WPV data could assist with the creation of local office–based initiatives specifically protecting US primary care clinicians. Highlighting the unique challenges that the primary care setting presents would allow for specific protections and federal support for programs to address WPV. For example, there has been some data to suggest that implementing risk assessment can reduce short term WPV. In one US study conducted at a Veterans Affairs hospital, flagging violent patients reduced violent incidents by 91.6%.^69^ Another study noted a significant reduction in violent attacks by 34% when using a short-term risk assessment followed by preventive measures in a psychiatric hospital.^70^

Additionally, when considering WPV, the unique experiences of male and female primary care clinicians should be individually considered. Historically, women have consistently been shown to be targets of sexual harassment from both their colleagues and by patients.^9,51,62^ Women represent a significant proportion of clinicians, including in adult primary care related fields. In 2019, women were 38.7% of internal medicine, 41.3% of family medicine/general practice, and 53.1% of medicine/pediatrics (Association 2020) This highlights the urgent need for more rigorous research into the effects of WPV on different genders in the primary care setting.

This narrative review on primary care WPV has several limitations. We reported mainly on smaller cross-sectional studies, and the lack of prospective studies weakened our ability to analyze patterns of violence over time in the primary care setting. Additionally, due to the lack of uniform definitions and terminology of WPV, it is possible that our literature search missed additional studies. Another significant limitation is the lack of data from the United States. While it is reasonable to extrapolate some general assumptions about WPV in primary care based on international studies, it is also possible that there are unique features of primary care WPV in the US, such as systemic barriers to access of healthcare, that may be uniquely relevant. Future research in the United States needs to begin with describing the scope of WPV in primary care settings. Such studies should focus not only on describing the frequency and type of violence committed, but also conduct root cause analyses to identify common health care driven causes as well as patient driven causes. We excluded studies conducted in primarily pediatric settings from this current review, though future studies that incorporate pediatric settings may enhance our current understanding of primary care WPV.

In summary, we found that the data on primary care WPV in the USA is scarce. International studies do provide some insight into this problem. WPV can take many forms but can generally be either physical or non-physical WPV. Non-physical WPV is the most reported type of WPV, with most of it being verbal. While both men and women experience physical and non-physical forms of WPV, women are consistently more likely to experience sexual harassment, and therefore, more study on these gender differences is necessary. There are numerous potential causes of WPV in primary care including those from the patient, the clinician, clinical-encounter based causes, and operational causes. Irrespective of the form or the root cause of WPV, it leaves a detrimental impact. Finally, more, higher quality studies are needed in the United States to better inform both local and national interventions to address this problem.

## Data Availability

All data generated or analyzed during this study are included in this published article [and its supplementary information files].
